# The Importance of Marital Status in the Morbidity and Prognosis of Lung Metastasis in Newly Diagnosed Ovarian Cancer

**DOI:** 10.7150/jca.83017

**Published:** 2023-04-09

**Authors:** Lihui Qian, Yixin Chen, Mingying Peng, Yuwei Xia, Tianye Zhou, Jiana Hong, Shuning Ding

**Affiliations:** 1 The First School of Clinical Medicine, Zhejiang Chinese Medical University, Hangzhou 310053, China.; 2 Department of Nursing, The First Affiliated Hospital of Zhejiang Chinese Medical University (Zhejiang Provincial Hospital of Traditional Chinese Medicine), Hangzhou 310006, China.

**Keywords:** lung metastasis, ovarian cancer, nomogram, SEER

## Abstract

**Objective:** The study aimed to evaluate the risk factors for the morbidity and prognosis of lung metastases (LM) in patients with newly diagnosed ovarian cancer (OC), and further explore the important role of marital status.

**Materials and methods:** Based on the Surveillance, Epidemiology, and End Results (SEER) dataset, OC patients from 2010 and 2019 were retrospectively analyzed. Logistic regression analysis and Kaplan-Meier method were applied to evaluate the vital factors of incidence and survival outcome in LM population. Cox regression analysis was performed to identify risk factors for the prognosis of OC patients with LM. The predictive potential was showed by two established nomograms and examined by the concordance index (C-index), calibration curves, the area under the curve (AUC), decision curve analyses (DCAs) and clinical impact curves (CICs).

**Results:** There are 25,202 eligible OC patients were enrolled in the study, the morbidity of LM at 5.61%. Multivariable logistic regression models illustrated that chemotherapy (*P<0.01*), surgical treatment of bilateral or more areas (*P<0.01*), T stage (*P<0.01*), N1 stage (*P<0.01*), bone metastasis (*P<0.01*), brain metastasis (*P<0.01*) and liver metastasis (*P<0.01*) were all significantly connected with LM in OC. Multivariable Cox regression analyses illustrated that unmarried, radiotherapy, elder people and positive cancer antigen 125 (CA-125) were significantly associated with shorter survival time, while chemotherapy made contributions to improve survival. Our study found that marital relationships promoted LM and was associated with the better prognosis, while unmarried patients had the opposite results. With the further development of our research, the cross-action of social, economic and psychological factors together determined the great impact of marital status on the morbidity and prognosis of OC patients combined with LM. Finally, the stability of the models was proved by internal verification.

**Conclusion:** The population-based cohort study provides references for guiding clinical screening and individualized treatment of OC patients with LM. Under the influence of society and economy, marital status is closely related to the morbidity and prognosis of OC, which can be an important direction to explore the risk of OC lung metastasis in the future.

## Introduction

Ovarian cancer (OC) is the most common cause of death in the female genital system. The American Cancer Society reported that there were 21,750 new cases of OC and 13,940 deaths in 2020^1^. Approximately 60% of OC patients were determined at an advanced stage. Previous studies reported that the invasion of OC cells mainly depends on the hematogenous circulation and lymphatic channels^2^, and pulmonary metastases in OC always are classified at a lower-level outcome that notably leads to a poor prognosis^3^, ^4^. As the second most common distant metastatic site, the morbidity of lung metastasis (LM) ranges from 6% to 16%^5^-^8^. Although surgery is preferred as the curative treatment for metastatic malignancies, a large number of affected people cannot be operated because of the strict indications^9^. OC patients with LM can also be beneficial from systemic treatments, including chemotherapy, radiotherapy, targeted therapy, and immunotherapy^10^-^12^. Although these treatments do prolong the progression-free survival, most patients would ultimately suffer from relapse or resistance^13^. Meanwhile, the huge economic burden also challenges them. It is necessary to explore risk factors for the morbidity and prognosis of OC patients with LM, thus enhancing the survival outcomes.

Through literature reviews, risk factors and survival estimates of OC patients with LM have not been Intensively analyzed. Therefore, it is essential to construct predictive models for designing prophylactic treatments and attentive nursing care for OC patients at a high risk of LM. This study aims to investigate risk factors for the morbidity and prognosis of newly diagnosed OC patients with LM and validate them by establishing nomograms.

## Materials and Methods

### Patient selection

**Figure 1:**
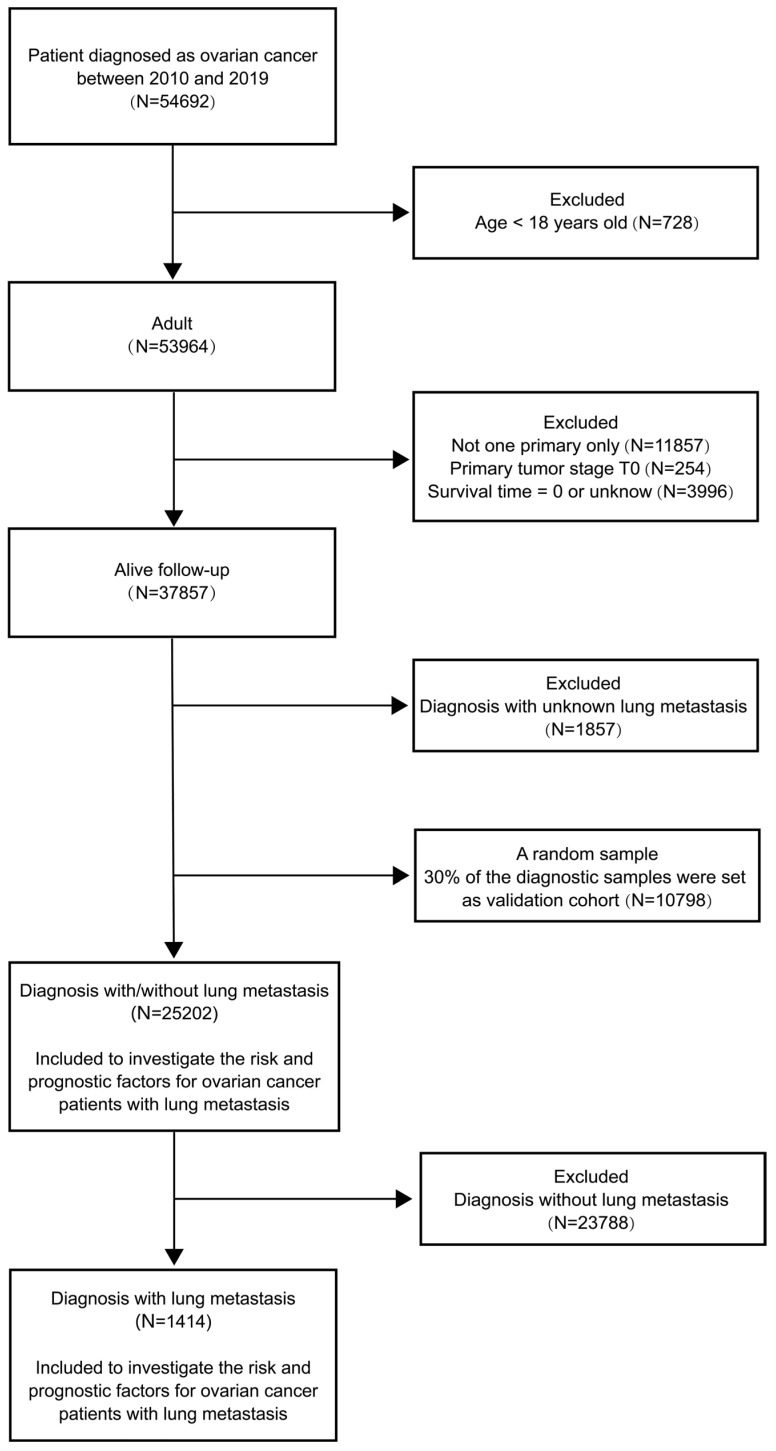
The flow diagram of participant inclusion and exclusion.

We searched and downloaded OC patients' medical records from the SEER Research Plus Data (17 Registries), which covered more than a third of the American population on cancer morbidity and survival records. A total of 36000 patients diagnosed as OC based on the Site and Morphology, TNM 7/CS v0204+ Schema recode from January 2010 to December 2019 were recruited in our study. We assigned 25,202 patients to the training cohort (70%) and 10,798 patients to the validation cohort (30%). The flow diagram of participant inclusion and exclusion was presented in Figure 1. The ethical approval was not requested in this study because clinical data of recruited OC patients were collected from the open-access and anonymized data in the public SEER dataset.

### Variable statement

The demographic and clinical characteristics mentioned were identified and prescribed as follows: year of diagnosis (2010-2012, 2013-2014, 2015-2016, 2017-2019), age at diagnosis (18-49, 50-69, 70-79, ≥ 80 years), race (black, white, other/not stated), marital status [married (including married, separated), unmarried (including single, divorced, widowed, unmarried or domestic partner), unknown], histology (non-serous, serous adenocarcinoma, other/not stated), grade (I = well differentiated, II = moderately differentiated, III = poorly differentiated, IV = undifferentiated, unknown), tumor size (< 50mm, 50-120mm, ≥ 120mm, not stated), examined regional nodes number (0, 1-10, ≥ 11, not stated), surgical treatments on the primary site (unilateral, bilateral/other, no/unknown), tumor phase (T1, T2, T3, not stated), regional lymph node phase (N0, N1, not stated), CA-125 (negative, positive, borderline/unknown), the usage of radiotherapy and chemotherapy, as well as the appearance of bone metastasis, brain metastasis, and liver metastasis.

### Nomogram construction and validation

In the cohort, the classified variables were expressed as the number and its percentage (N, %). Follow-up analyses were conducted to assess independent risks for the prognosis of OC with LM. Univariable and multivariable logistic regression models were introduced to identify risk factors for the morbidity of LM in newly diagnosed OC patients, and adjusted and unadjusted proportional hazard models were used to distinguish prognostic factors for OC with LM. Two nomograms were constructed in R based on multivariable logistic regression, multivariable Cox regression, and potential risk factors (P < 0.05) in the training cohort using the rms package. The predictive performance of the nomograms was measured by the C-index. Based on risk scores of overall survival (OS) in the nomogram, patients were categorized into low-risk and high-risk subgroups. Differences between two subgroups were assessed by depicting the clinic effect curve. Furthermore, Kaplan-Meier survival curves were depicted to assess the overall survival of OC patients with LM. To avoid the impact of other critical illnesses, cancer-specific survival analyses were performed by the cumulative incidence function. Notably, the accuracy of nomogram was detected and validated by operating calibration plots. Also, DCAs and CICs were designed to calculate the net avails for each risk threshold probability.

### Statistical analysis

R software (version 4.2.1) was employed for statistical analyses. The categorical data were measured by Fisher's exact test or Chi-square test. Propensity score matching (PSM) analysis was used to unify the baseline of all covariates in patients with and without LM. The 1:4 nearest neighbor matching method was adopted with a caliper value set at 0.2. The matching results showed the differences among the whole clinical parameters. Nomograms based on regression models, calibration curves and survival-related curves were all drawn via diverse functional packages namely RMS, Foreign, Survival, Cmprsk and other software. A two-tailed *P* value *< 0.05* was considered as statistical significance (*P < 0.05, ** P < 0.01).

## Results

### Baseline characteristics of OC patients

As shown in Table 1, a total of 25,202 eligible OC patients were recruited in the training cohort. Their median survival time were 28 months (interquartile range 11-56 months), respectively. Among them, 5.61% (N = 1,414) developed LM with the14 months (interquartile range 4-29 months) median survival time, respectively. Other demographic and medical traits of recruited OC patients were presented as well. The remarkable differences included the age, race, marital status, histology, grade, tumor size, the number of regional nodes examination, radiotherapy, chemotherapy, surgery scope, T stage, N stage, CA-125, bone metastasis, brain metastasis and liver metastasis. In the results of PSM analysis, 1398 patients were matched in the LM group and 5060 patients were actually matched in the without LM group. There were statistically significant differences in baseline characteristics between the two groups, including histology, grade, regional nodes examined, surgery scope, T stage, N stage, CA-125, bone metastasis, brain metastasis, and liver metastasis. The baseline characteristics of the validation cohort were shown in Table 2.

### Independent risk factors for the morbidity of LM in OC patients and nomogram establishment

Based on the results of chi-square test and PSM analysis (Table 1), unadjusted and adjusted logistic regression analyses were applied to assess independent risk factors for the morbidity of LM in newly diagnosed OC patients. It was shown that the histology, grade, the number of examined regional nodes, treatment strategies like chemotherapy and surgery, T and N stage, CA-125, and the incidence of other distant metastases were correlated with the morbidity of LM in OC patients (Table 3). The morbidity of LM in OC patients with the histological subtype of serous adenocarcinoma was significantly lower than those with non-serous adenocarcinoma (OR=0.84, 95%CI=0.73-0.97, *P<0.01*). Concerning tumor grade, poorly differentiated (OR=3.68, 95%CI=1.84-8.78, *P<0.01*), and undifferentiated OC patients (OR=3.66, 95%CI=1.81-8.75, *P<0.01*) had a significantly higher risk of LM development than well differentiated ones. In addition, OC patients with more than 10 examined lymph nodes had a significantly lower risk for the morbidity of LM than those without lymph nodes detection (OR=0.43, 95%CI=0.34-0.54, *P<0.01*). Advanced T and N stage, especially T3 stage (OR=2.49, 95%CI=1.91-3.29, *P<0.01*) and N1 stage (OR=1.90, 95%CI=1.64-2.20, *P<0.01*) were risk factors for LM development in OC patients. Patients with surgical treatment of bilateral ovaries or more areas had a higher risk of LM development than those receiving unilateral ovary surgery (OR=1.45, 95%CI=1.18-1.80, *P<0.01*), which might be attributed to disease development itself. And patients with positive CA-125 (OR=2.22, 95%CI=1.51-3.42, *P<0.01*) had a significantly higher risk of LM than negative ones. Besides, chemotherapy (OR=1.50, 95%CI=1.28-1.75, *P<0.01*), bone metastasis (OR=3.33, 95%CI=2.44-4.51, *P<0.01*), brain metastasis (OR=5.72, 95%CI=3.19-10.28, *P<0.01*) and liver metastasis (OR=3.64, 95%CI=3.17-4.18, *P<0.01*) were all risk factors for the morbidity of LM in OC patients.

**Figure 2:**
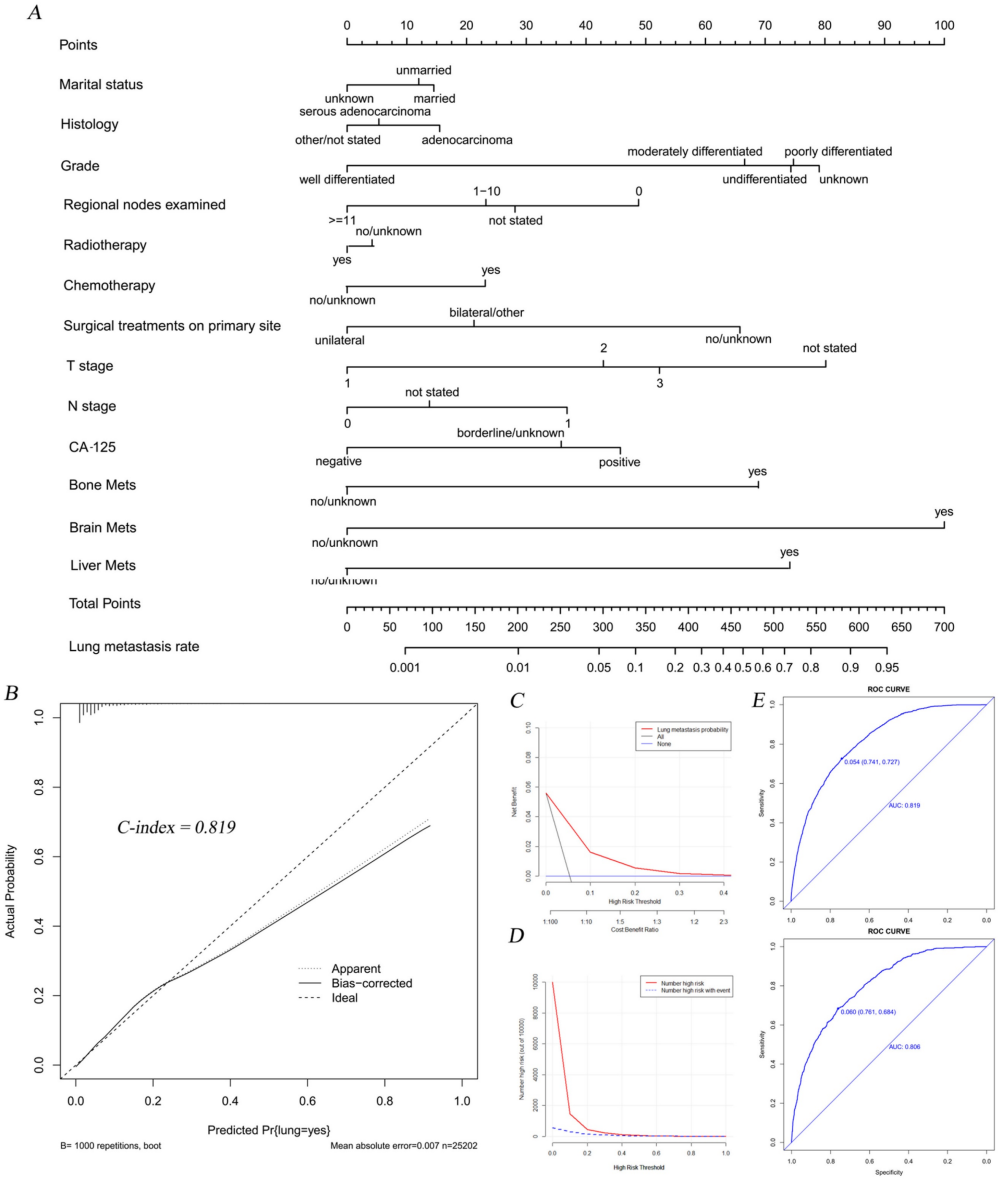
The nomogram combining with its calibration and verification curves for predicting LM morbidity in OC patients. A total of thirteen factors were determined in LM incidence predictive nomogram (A). The calibration curve (B) with the C-index of 0.819 was showed to verify the validity of prediction. Decision curve (C) and clinical impact curve (D) were plotted to show the event occurrence of patients with high risks. The calibration curves (E) with similar values of AUC (Training cohort AUC=0.819, verification cohort AUC=0.806) showed good predictability of the model.

Subsequently, we established a nomogram to intuitively display score assignments and predictive probability of the risk factors (Figure 2A). Simultaneously, the calibration curve with the C-index of 0.819 suggested an extremely consistency between actual observations and the probability of prediction (Figure 2B). DCAs and CICs illustrated that threshold probabilities at 0-0.3 were the most favorable predictor of LM in accordance with our nomogram model (Figure 2C-D). The calibration curve with similar AUC values showed good predictability of our nomogram model (Figure 2E).

### Survival analyses of OC patients with LM

**Figure 3:**
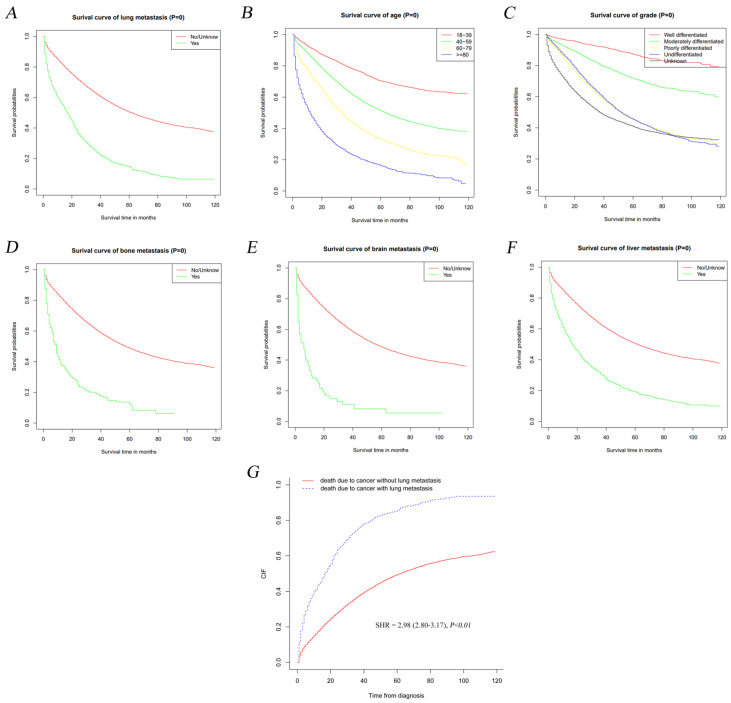
Kaplan-Meier analyses of OS among populations who were diagnosed with lung metastasis relative to the other (A) and stratified by age (B), grade (C), bone metastasis (D), brain metastasis (E) and liver metastasis (F) in ovarian cancer. And ovarian cancer-specific survival curve (G) in ovarian cancer.

Kaplan-Meier method was adopted to detect the influence of LM on the outcome of OC patients. As shown in Figure 3A, OS curves revealed that LM development was significantly correlated to the prognosis of OC (*P<0.01*). The OS was significantly worse in OC patients with over 80 years of age (Figure 3B, *P<0.01*), poorly differentiated and undifferentiated neoplasm (Figure 3C, *P<0.01*), bone metastasis (Figure 3D, *P<0.01*), brain metastasis (Figure 3E, *P<0.01*) and liver metastasis (Figure 3F, *P<0.01*) than those of controls. Meanwhile, we found that LM was significantly correlated with the major cause of death in OC patients rather than other diseases via Gray method [sub-distribution hazard ratio (SHR)=2.98, 95%CI=2.80-3.17, *P<0.01*] (Figure 3G).

### Prognostic factors for OC and nomogram establishment

Based on the results of chi-square (Table 1), prognostic factors for OC patients were analyzed using the Cox regression model (Table 4). Univariable Cox regression model showed fifteen factors closely related to the occurrence of lung metastasis. Multivariable Cox regression model showed that OC patients over 80 years (HR=1.34, 95%CI=1.05-1.71, *P=0.02*) were detected to have a higher risk of death. Concerning treatment strategies, a lower risk of death was detected in OC patients treated with chemotherapy (HR=0.36, 95%CI=0.31-0.43, *P<0.01*). Grade was a significant risk factor for the prognosis of OC, especially Moderately differentiated (HR=3.96, 95%CI=1.21-12.96, *P=0.02*). Likewise, positive CA-125 (HR=1.65, 95%CI=1.03-2.66, *P=0.04*), bone metastasis (HR=1.22, 95%CI=0.95-1.56, *P<0.01*), brain metastasis (HR=1.91, 95%CI=1.27-2.88, *P=0.12*) and liver metastasis (HR=1.25, 95%CI=1.09-1.42, *P<0.01*) were all Significant independent risk factors for the prognosis of OC.

**Figure 4:**
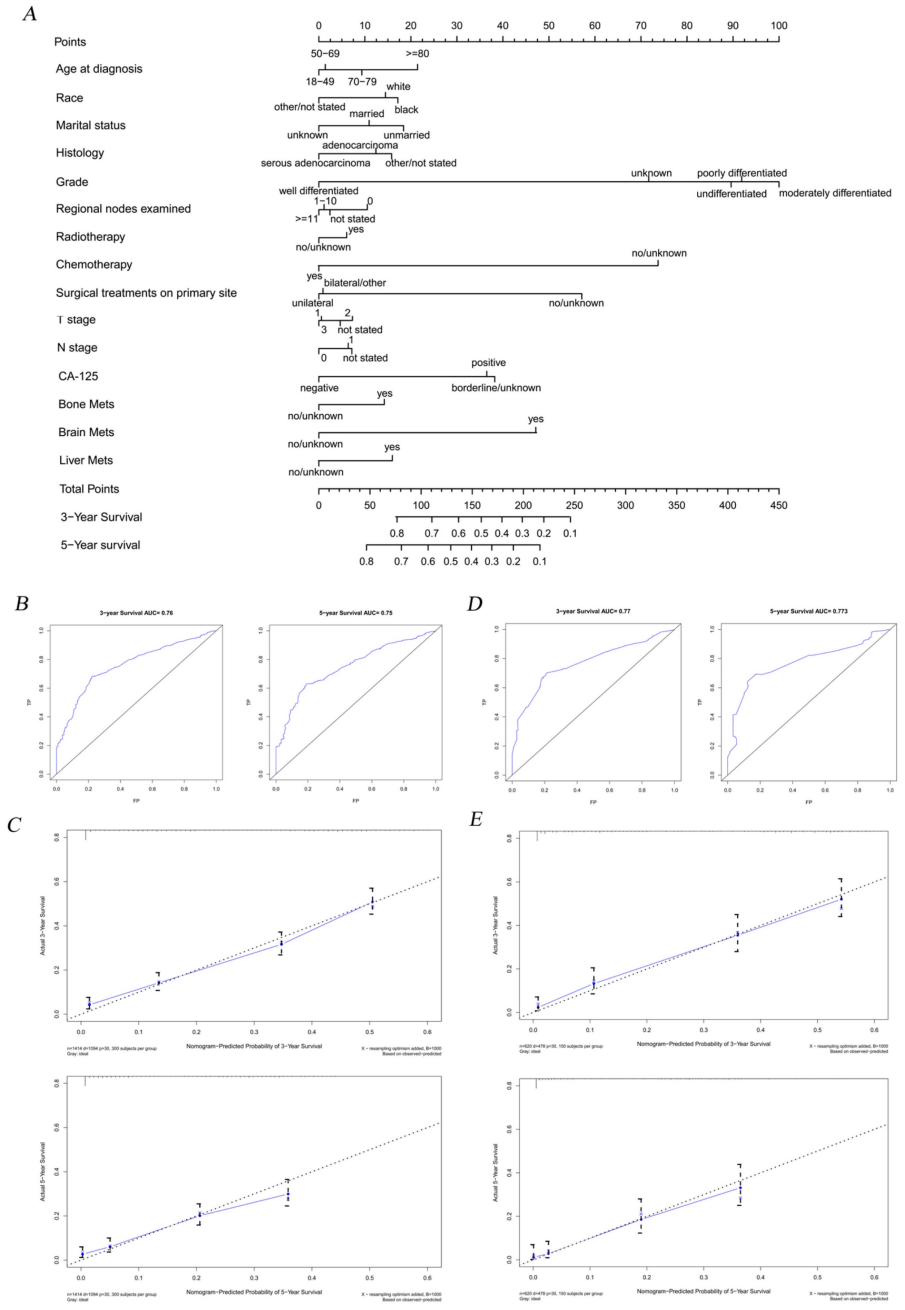
The nomogram combining with its calibration and verification curves for predicting risk factors on prognosis for OC patients. A total of fifteen prognostic factors were defined in 3- and 5-year survival nomogram (A). Calibration curves of training cohort (B-C) with the values of AUC (3-year AUC=0.76, 5-year AUC=0.75, respectively) were plotted to verify the effectiveness of prediction. Calibration curves of verification cohort (D-E) with similar AUC values demonstrate the accuracy of the prediction model.

**Figure 5:**
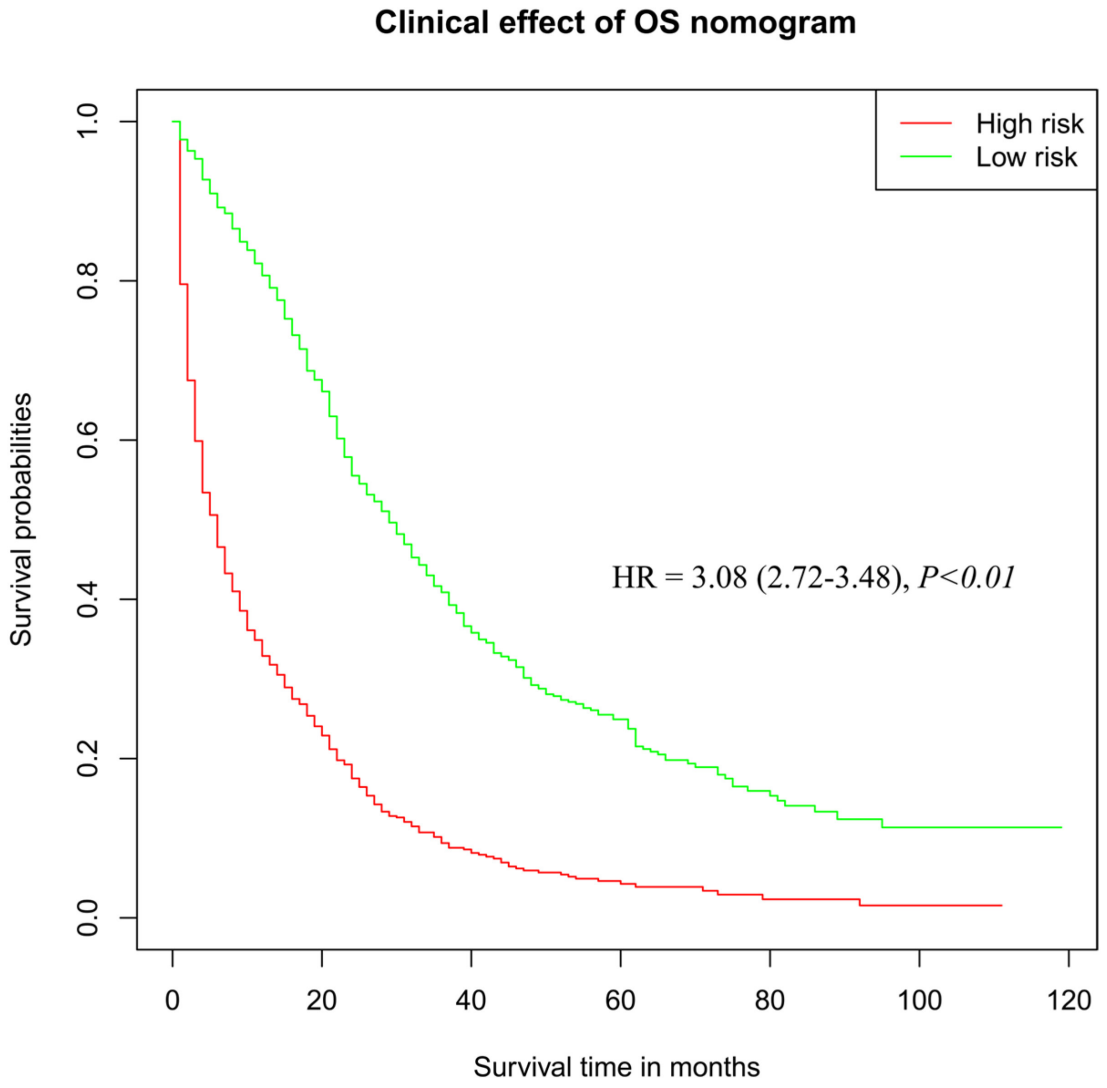
The clinical effect curve drawn from risk scores in the OS nomogram.

According to the results of Cox regression analysis, significant risk factors for the prognosis of OC were subjected to the establishment of a nomogram for determining the 3-year and 5-year survival rate (Figure 4A). Stratified by the medium scores from the nomogram, the clinic effect curve revealed that the high survival feasibility of low-risk subgroup was significantly superior to that of high-risk subgroup (Figure 5, HR=3.08, 95%CI=2.72-3.48, *P<0.01*). Furthermore, the calculated 3-year and 5-year AUC (0.76 and 0.75, respectively, Figure 4B) and the solid lines closed to the diagonal lines (Figure 4C) both displayed the excellent accuracy of the prediction. Calibration curves of verification cohort (Figure 4D-E) with similar AUC values demonstrated the accuracy of the prediction model.

## Discussion

Ovarian carcinoma is regarded as the first leading cause of mortality among gynecological malignancies due to its high recurrence rate and bad prognosis. Although risk factors for the prognosis of metastatic OC have been previously explored, we come up with new insights.

Previous studies listed some hazard elements for the morbidity and prognosis of OC with distant metastases, but the dated population data and the fuzzy visualizations were unconvincing^14^-^16^. Yuan et al.^17^ revealed that the advanced T and N stages and other distant metastases were risk factors for the morbidity of LM in OC patients, as well as active surgery and chemotherapy served as protective factors. Cao et al.^18^ and Xu et al.^19^ focused on the analysis of serous ovarian cancer and epithelial ovarian cancer, respectively. However, the results acquired from above studies were not comprehensive. Not only did we study the impacts of pathological types and some common factors on the occurrence and prognosis of OC, but also applied the new well-concerned chart form to improve the efficiency of clinical applications and better visualize the results. Meanwhile, we further excavated the influence of marital status on the survival outcome of OC patients, and made the explanation on the contradiction between chemotherapy and radiotherapy in the two nomograms. The high accuracy and stability of our prediction models were evaluated by AUC, C-index and excellent internal validation results.

According to the cohort analyses, 5.61% of the included OC patients were diagnosed with LM and the median overall survival was 14 months. We found that OC patients with a high tumor grade, pathological types of non-serous adenocarcinoma, the intervention of chemotherapy, higher level TNM stage, positive CA-125, and other organ metastases were likely to develop LM. As for prognosis, older age, moderate grade, lack of regional lymph node examination, radiotherapy treatment, elevated CA-125, T2 and N1 phases and distant metastases were found to be significantly related. What's more, we have verified the high precision of nomograms with a series of methods containing the C-index, calibration plots as well as the value of AUC, which all demonstrated the high agreement with the accuracy. CA-125 is a large membrane glycoprotein, belonging to the wide mucin family. Thirty years after its discovery, CA-125 is still recommended as a vital tumor marker, which is detected to reflect cancer cell residue or recurrence in OC patients after the first-line therapy^20^. It is proved that a rising serum CA-125 level within the normal range is strongly associated with recurrence risk and survival outcome of OC^21^, suggesting that the fluctuated CA-125 level is valuable for predicting the prognosis of OC.

According to previous investigations, the serous adenocarcinoma is considered as the most aggressive subtype^22^, while our results showed that the non-serous adenocarcinoma was more correlated with the development of LM. More evidences revealed that smoke exposure increased the number of lung nodules^23^, ^24^, and enhanced the risk of non-serous carcinomas, especially mucinous tumors^25^, ^26^, which might explain the reason why the pathological type of non-serous adenocarcinoma accelerated LM. A previous study suggested that higher tumor grade and T stage were crucial risk factors for the prognosis of gynecological cancer patients with distant metastases^27^. Not surprisingly, we obtained the similar result that undifferentiation and poor differentiation grade, worse T and N stages and lack of regional node examination were significantly correlated with the risk of OC with LM. In addition, we found that patients with OC who underwent bilateral surgery had a higher risk of lung metastasis, most likely because the underlying cancer cells had already completed distant metastasis before surgery. Cancer cells in both ovaries are theoretically at higher risk of distant metastasis and spread than those in one ovary. Obviously, the survival probability in low-risk subgroup was dramatically higher than that in high-risk OC patients, indicating that identifying risk factors was instructive and meaningful for guiding prophylactic clinical treatment and improving the prognosis of OC patients. In this study, we not only evaluated the impact of these factors, but also calculated the cancer-specific survival by the methods of eliminating the intervention from other diseases. The clinic effect curve showed the discrimination ability of models.

As for OS nomogram, we revealed that the prognosis of younger OC patients aging18-49 years was better than older ones, which was consistent with previous findings^28^, ^29^. After all, it's inevitable that bodily functions decline significantly with age. In terms of race, blacks had higher mortality rates and preferred to a refusal of adjuvant chemotherapy after surgery compared to whites, as our results showed^30^. It is reported that an elevated CA-125 level indicated an ineffective treatment^31^. Likewise, our study found that elevated CA-125 level resulted in worse survival outcomes, which was recognized as an effective determinant for the prognosis of OC with LM. In addition, we found that three lower differentiation grades were extremely detrimental to survival compared to well differentiated grades but there were less differences from each other. Both surgery and chemotherapy were the positive elements in increasing life expectancy for cancer patients with regional lymph node involvement^32^, which was also proved by our Cox regression analyses. Of note, the assessment of risk factors and biomarkers at the cellular level for chemotherapy response should be highlighted in the future, especially for relapsing population or patients with high risk factors^33^.

Interestingly, we found that the impact of marital status on OC patients was worthy of further investigations. In the SEER Research Plus Data, seven different marital statuses are recorded. Considering multiple psychological and economic factors influenced by legal references and societal norms, we classified "married (including common law)" and "separated" as Married group, and "single (never married)," "divorced," "widowed," "unmarried or domestic partner" as Unmarried group. Our study demonstrated that married OC patients tended to develop LM but had a better prognosis than unmarried population. This phenomenon has been explained by different sociologists. Studies have shown that divorce/widower and low social integration are chronically psychosocial stressors that may affect health. In the social model made by Trudel-Fitzgerald et al., social isolation is regarded as an independent risk factor for OC patients, which is as important as some traditional determinants (e.g., family history of breast/ovarian cancer, history of hormone therapy)^34^. Wang et al.^35^ proposed that marriage could acquire more family emotional support and better economic conditions, which contributed to increase patients' confidence in fighting the disease and improving patients' compliance. Gardner et al.^36^ also agreed with this argument and arguing that marriage was beneficial to a strict adherence to standard chemotherapy care. The emotional state of comfort, happiness and pleasure that marriage brings was also beneficial to the construction of a healthy mental environment^37^. In addition, Gardner's work told us that married adults and their spouses in the United States were much easier to be insured than single people, including unmarried and divorced people. In another study involving race and socioeconomic relations, Bristow et al.^38^ reported that the uninsured patients generally rejected treatment that meet the National Comprehensive Cancer Network's guidelines. Above all, marriage plays an essential role in social relations and medical economy, which is closely connected with prognostic outcomes in OC patients. However, it is not yet known whether marital status has an exact effect on distant metastasis of cancer, which provides new ideas for future research.

The applications of radiotherapy and chemotherapy brought out opposite effects in two nomograms. Current data demonstrated that chemotherapy was feasible for partial cytoreduction and prolonged survival^39^, ^40^, while chemotherapy resistance also resulted in the recurrence and metastases of cancer^41^. It is undeniable that chemotherapy, as the primary treatment for most OC patients, can significantly improve clinical response and outcome, which is also the reason why patients with advanced OC had chemotherapy treatment experiences^42^, ^43^. Furthermore, radiotherapy is beneficial to immune regulation and reconstruction of the tumor microenvironment. Palliative radiotherapy made great contribution to relieving pain and bleeding, and reducing the abdominal mass. At the same time, the toxic side effects of radiotherapy to accelerate the risk of poor prognosis cannot be ignored^16^, ^44^. Originally, the survival expectation of OC patients participating in palliative radiotherapy is not optimistic, which also explain the poor prognosis of non-chemotherapy patients in OS nomogram.

However, our study still had several limitations. Firstly, this population-based retrospective investigation lacked some pivotal clinical data, such as the detailed assessment about pulmonary metastatic tumors and more information on individual treatments. Secondly, the obtained morbidity of LM might produce regional biases since the model was built based on registered data from the United States. Last but not the least, it would be better if the external validation was added in the study.

## Conclusions

The retrospective study represented the largest dataset for LM development in OC patients and provided valuable nomograms about epidemiological characteristics and prognosis of advanced OC. Moreover, our findings suggested a strong reliability through multiple statistic approaches of calibration and discrimination. Hence, they had the potential to guide clinical diagnosis and individual treatments of OC with LM. In the future, laboratory investigations and large sample prospective clinical trials are demanded to further evaluate the molecular characteristics and treatment decisions for OC patients with LM.

## Author contributions

LQ and YC designed, recorded and compiled the data. MP, YX and TZ wrote the manuscript. JH and SD organized, conceived, and supervised the research. The whole authors participated and approved the manuscript.

## Availability of data and materials

All the records generated for this research is available in the SEER dataset (https://seer.cancer.gov/about/overview.html).

## Ethics approval and consent to participate

This study depended on publicly available de-identified data from the SEER dataset that did not involve the use of personally identifiable information or interaction with the included population. The informed consent from the SEER registered cases in this study was not necessary and the authors obtained Limited-Use Data Agreements from SEER. No trial registration was required.

## Figures and Tables

**Table 1:** Clinicopathological traits for OC patients diagnosed with and without lung metastasis.

Subject Characteristics	Number of ovarian cancer patients in the training cohort				PSM (1:4)			
	With LM (N=1414, 5.61%)	Without LM (N=23788, 94.39%)	X^2^	*P*	With LM (N=1398, 21.6%)	Without LM (N=5060, 78.4%)	X^2^	*P*
**Year of diagnosis**			6.43	0.09			5.67	0.13
2010-2012	443 (0.31)	7067 (0.30)			441 (0.32)	1607 (0.32)		
2013-2014	275 (0.19)	4887 (0.21)			272 (0.19)	1077 (0.21)		
2015-2016	320 (0.23)	4961 (0.21)			317 (0.23)	1013 (0.20)		
2017-2019	376 (0.27)	6873 (0.29)			368 (0.26)	1363 (0.27)		
**Age at diagnosis**			108.22	<0.01			4.60	0.20
18-49	180 (0.13)	5137 (0.22)			176 (0.13)	697 (0.13)		
50-69	684 (0.48)	11994 (0.50)			677 (0.48)	2364 (0.47)		
70-79	334 (0.24)	4209 (0.18)			332 (0.24)	1139 (0.23)		
≥80	216 (0.15)	2448 (0.10)			213 (0.15)	860 (0.17)		
**Race**			19.77	<0.01			2.83	0.24
Black	164 (0.12)	1971 (0.08)			161 (0.11)	530 (0.10)		
White	1101 (0.78)	19014 (0.80)			1090 (0.78)	4049 (0.80)		
Other/not stated	149 (0.11)	2803 (0.12)			147 (0.11)	481 (0.10)		
**Marital status**			12.41	<0.01			2.49	0.29
Married	664 (0.47)	12166 (0.51)			660 (0.47)	2426 (0.48)		
Unmarried	697 (0.49)	10592 (0.45)			685 (0.49)	2399 (0.47)		
Unknown	53 (0.04)	1030 (0.04)			53 (0.04)	235 (0.05)		
**Histology**			21.70	<0.01			7.34	0.02
Adenocarcinoma	445 (0.32)	6804 (0.29)			434 (0.31)	1398 (0.28)		
Serous adenocarcinoma	701 (0.50)	13248 (0.56)			699 (0.50)	2716 (0.54)		
Others/not stated	268 (0.19)	3736 (0.16)			265 (0.19)	946 (0.19)		
**Grade**			242.88	<0.01			11.65	0.02
Well differentiated	7 (0.01)	1495 (0.06)			7 (0.01)	72 (0.01)		
Moderately differentiated	46 (0.03)	2019 (0.08)			46 (0.03)	177 (0.03)		
Poorly differentiated	279 (0.20)	5569 (0.23)			279 (0.20)	955 (0.19)		
Undifferentiated	193 (0.14)	4197 (0.18)			193 (0.14)	794 (0.16)		
Unknown	889 (0.63)	10508 (0.44)			873 (0.62)	3062 (0.61)		
**Tumor size**			386.03	<0.01			7.70	0.05
<50mm	160 (0.11)	3903 (0.16)			159 (0.11)	535 (0.11)		
50-120mm	350 (0.25)	7628 (0.32)			349 (0.25)	1323 (0.26)		
>120mm	199 (0.14)	6037 (0.25)			194 (0.14)	831 (0.16)		
Not stated	705 (0.50)	6220 (0.26)			696 (0.50)	2371 (0.47)		
**Regional nodes examined**			396.82	<0.01			8.94	0.03
0	1061 (0.75)	11860 (0.50)			1045 (0.75)	3673 (0.73)		
1-10	183 (0.13)	5568 (0.23)			183 (0.13)	735 (0.15)		
11+	103 (0.07)	5618 (0.24)			103 (0.07)	461 (0.09)		
Not stated	67 (0.05)	742 (0.03)			67 (0.05)	191 (0.04)		
**Radiotherapy**			26.98	<0.01			2.51	0.11
No/unknown	1374 (0.97)	23499 (0.99)			1362 (0.97)	4964 (0.98)		
Yes	40 (0.03)	289 (0.01)			36 (0.03)	96 (0.02)		
**Chemotherapy**			20.62	<0.01			0.07	0.79
No/unknown	300 (0.21)	6350 (0.27)			296 (0.21)	1055 (0.21)		
Yes	1114 (0.79)	17438 (0.73)			1102 (0.79)	4005 (0.79)		
**Sur (pri)**			1144.76	<0.01			26.80	<0.01
Unilateral	112 (0.08)	6384 (0.27)			112 (0.08)	347 (0.07)		
Bilateral or more area	568 (0.40)	13454 (0.57)			567 (0.41)	2447 (0.48)		
No/unknown	734 (0.52)	3950 (0.17)			719 (0.51)	2266 (0.45)		
**T stage**			784.06	<0.01			13.73	<0.01
T1	69 (0.05)	6761 (0.28)			68 (0.05)	277 (0.05)		
T2	125 (0.09)	2980 (0.13)			125 (0.09)	351 (0.07)		
T3	803 (0.57)	11734 (0.49)			801 (0.57)	3119 (0.62)		
Not stated	417 (0.29)	2313 (0.10)			404 (0.29)	1313 (0.26)		
**N stage**			367.65	<0.01			34.44	<0.01
N0	616 (0.44)	16140 (0.68)			610 (0.44)	2510 (0.50)		
N1	449 (0.32)	4717 (0.20)			447 (0.32)	1228 (0.24)		
Not stated	349 (0.25)	2931 (0.12)			341 (0.24)	1322 (0.26)		
**CA-125**			116.63	<0.01			18.71	<0.01
Negative	26 (0.02)	2354 (0.10)			26 (0.02)	203 (0.04)		
Positive	1119 (0.79)	16331 (0.69)			1107 (0.79)	3800 (0.75)		
Borderline/unknown	269 (0.19)	5103 (0.21)			265 (0.19)	1057 (0.21)		
**Bone Mets**			474.00	<0.01			31.75	<0.01
No/unknown	1322 (0.93)	23634 (0.99)			1318 (0.94)	4925 (0.97)		
Yes	92 (0.07)	154 (0.01)			80 (0.06)	135 (0.03)		
**Brain Mets**			244.63	<0.01			17.61	<0.01
No/unknown	1380 (0.98)	23752 (0.99)			1373 (0.98)	5029 (0.99)		
Yes	34 (0.02)	36 (0.01)			25 (0.02)	31 (0.01)		
**Liver Mets**			1280.95	<0.01			38.07	<0.01
No/unknown	998 (0.71)	22554 (0.95)			997 (0.71)	4003 (0.79)		
Yes	416 (0.29)	1234 (0.05)			401 (0.29)	1057 (0.21)		

**Abbreviations**: LM=lung metastasis; Sur (pri)=surgical treatments on primary site; CA-125=cancer antigen 125; Mets=metastasis.

**Table 2:** Clinicopathological traits for validation cohort.

Subject Characteristics	Number of ovarian cancer patients in the validation cohort				PSM (1:4)			
	With LM (N=620, 5.74%)	Without LM (N=10178, 94.26%)	X^2^	*P*	With LM (N=603, 21.7%)	Without LM (N=2175, 78.3%)	X^2^	*P*
**Year of diagnosis**			0.27	0.97			0.24	0.97
2010-2012	184 (0.30)	2973 (0.29)			179 (0.30)	652 (0.30)		
2013-2014	123 (0.20)	2092 (0.21)			119 (0.20)	445 (0.20)		
2015-2016	136 (0.22)	2183 (0.21)			130 (0.22)	458 (0.21)		
2017-2019	177 (0.29)	2930 (0.29)			175 (0.29)	620 (0.29)		
**Age at diagnosis**			50.41	<0.01			0.29	0.96
18-49	80 (0.13)	2220 (0.22)			76 (0.13)	292 (0.13)		
50-69	304 (0.49)	5151 (0.51)			298 (0.49)	1060 (0.49)		
70-79	134 (0.22)	1782 (0.18)			129 (0.21)	464 (0.21)		
≥80	102 (0.16)	1025 (0.10)			100 (0.17)	359 (0.17)		
**Race**			6.03	0.05			1.82	0.40
Black	69 (0.11)	849 (0.08)			68 (0.11)	212 (0.10)		
White	480 (0.77)	8187 (0.80)			466 (0.77)	1734 (0.80)		
Other/not stated	71 (0.11)	1142 (0.11)			69 (0.11)	229 (0.11)		
**Marital status**			5.49	0.06			0.52	0.77
Married	284 (0.46)	5152 (0.51)			277 (0.46)	1032 (0.47)		
Unmarried	310 (0.50)	4617 (0.45)			300 (0.50)	1046 (0.48)		
Unknown	26 (0.04)	409 (0.04)			26 (0.04)	97 (0.04)		
**Histology**			9.96	<0.01			1.67	0.43
Adenocarcinoma	202 (0.33)	2886 (0.28)			192 (0.32)	661 (0.30)		
Serous adenocarcinoma	307 (0.50)	5700 (0.56)			303 (0.50)	1156 (0.53)		
Others/not stated	111 (0.18)	1592 (0.16)			108 (0.18)	358 (0.16)		
**Grade**			99.12	<0.01			5.95	0.20
Well differentiated	7 (0.01)	669 (0.07)			7 (0.01)	44 (0.02)		
Moderately differentiated	17 (0.03)	902 (0.09)			16 (0.03)	96 (0.04)		
Poorly differentiated	119 (0.19)	2298 (0.23)			117 (0.19)	409 (0.19)		
Undifferentiated	94 (0.15)	1818 (0.18)			94 (0.16)	339 (0.16)		
Unknown	383 (0.62)	4491 (0.44)			369 (0.61)	1287 (0.59)		
**Tumor size**			130.23	<0.01			7.99	0.05
<50mm	87 (0.14)	1667 (0.16)			87 (0.14)	296 (0.14)		
50-120mm	157 (0.25)	3204 (0.31)			155 (0.26)	598 (0.27)		
>120mm	89 (0.14)	2665 (0.26)			84 (0.14)	390 (0.18)		
Not stated	287 (0.46)	2642 (0.26)			277 (0.46)	891 (0.41)		
**Regional nodes examined**			185.45	<0.01			14.10	<0.01
0	472 (0.76)	5066 (0.50)			458 (0.76)	1525 (0.70)		
1-10	72 (0.12)	2383 (0.23)			72 (0.12)	369 (0.17)		
11+	47 (0.08)	2407 (0.24)			46 (0.08)	211 (0.10)		
Not stated	29 (0.05)	322 (0.03)			27 (0.04)	70 (0.03)		
**Radiotherapy**			59.58	<0.01			1.42	0.23
No/unknown	593 (0.96)	10081 (0.99)			584 (0.97)	2125 (0.98)		
Yes	27 (0.04)	97 (0.01)			19 (0.03)	50 (0.02)		
**Chemotherapy**			7.35	<0.01			0.04	0.85
No/unknown	138 (0.22)	2772 (0.27)			135 (0.22)	479 (0.22)		
Yes	482 (0.78)	7406 (0.73)			468 (0.78)	1696 (0.78)		
**Sur (pri)**			518.87	<0.01			23.75	<0.01
Unilateral	51 (0.08)	2771 (0.27)			51 (0.08)	139 (0.06)		
Bilateral or more area	246 (0.40)	5749 (0.56)			243 (0.40)	1119 (0.51)		
No/unknown	323 (0.52)	1658 (0.16)			309 (0.51)	917 (0.42)		
**T stage**			297.75	<0.01			4.70	0.20
T1	34 (0.05)	2925 (0.29)			34 (0.06)	154 (0.07)		
T2	45 (0.07)	1221 (0.12)			45 (0.07)	149 (0.07)		
T3	370 (0.60)	4995 (0.49)			361 (0.60)	1361 (0.63)		
Not stated	171 (0.28)	1037 (0.10)			163 (0.27)	511 (0.23)		
**N stage**			165.76	<0.01			13.45	<0.01
N0	271 (0.44)	6956 (0.68)			270 (0.45)	1118 (0.51)		
N1	194 (0.31)	1955 (0.19)			183 (0.30)	508 (0.23)		
Not stated	155 (0.25)	1267 (0.12)			150 (0.25)	549 (0.25)		
**CA-125**			48.58	<0.01			10.71	<0.01
Negative	12 (0.02)	1008 (0.10)			12 (0.02)	102 (0.05)		
Positive	485 (0.78)	6955 (0.68)			474 (0.79)	1607 (0.74)		
Borderline/unknown	123 (0.20)	2215 (0.22)			117 (0.17)	466 (0.21)		
**Bone Mets**			323.84	<0.01			15.95	<0.01
No/unknown	570 (0.92)	10118 (0.99)			569 (0.94)	2122 (0.98)		
Yes	50 (0.08)	60 (0.01)			34 (0.06)	53 (0.02)		
**Brain Mets**			39.50	<0.01			0.63	0.43
No/unknown	613 (0.99)	10168 (0.99)			599 (0.99)	2166 (0.99)		
Yes	7 (0.01)	10 (0.01)			4 (0.01)	9 (0.01)		
**Liver Mets**			386.24	<0.01			11.43	<0.01
No/unknown	465 (0.71)	9649 (0.95)			461 (0.76)	1795 (0.83)		
Yes	155 (0.29)	529 (0.05)			142 (0.24)	380 (0.17)		

**Abbreviations**: LM=lung metastasis; Sur (pri)=surgical treatments on primary site; CA-125=cancer antigen 125; Mets=metastasis.

**Table 3:** The risk factors for lung metastasis in OC by logistic regression analyses.

Subject Characteristics	Univariable		Multivariable	
	OR (95%Cl)	P-value	OR (95%Cl)	P-value
**Marital status**				
Married	Reference		Reference	
Unmarried	1.21(1.08-1.35)	<0.01	0.96(0.85-1.08)	0.47
Unknown	0.94(0.70-1.24)	0.69	0.78(0.56-1.05)	0.10
**Histology**				
Adenocarcinoma	Reference		Reference	
Serous adenocarcinoma	0.81(0.72-0.91)	<0.01	0.84(0.73-0.97)	<0.01
Others/not stated	1.10(0.94-1.28)	0.25	0.76(0.64-0.91)	<0.01
**Grade**				
Well differentiated	Reference		Reference	
Moderately differentiated	4.87(2.34-11.83)	<0.01	3.19(1.51-7.87)	<0.01
Poorly differentiated	10.70(5.45-25.12)	<0.01	3.68(1.84-8.78)	<0.01
Undifferentiated	9.82(4.98-23.13)	<0.01	3.66(1.81-8.75)	<0.01
Unknown	18.07(9.29-42.21)	<0.01	3.97(1.99-9.42)	<0.01
**Regional nodes examined**				
0	Reference		Reference	
1-10	0.37(0.31-0.43)	<0.01	0.64(0.53-0.77)	<0.01
11+	0.20(0.17-0.25)	<0.01	0.43(0.34-0.54)	<0.01
Not stated	1.01(0.77-1.30)	0.94	0.70(0.52-0.92)	<0.01
**Radiotherapy**				
No/unknown	Reference		Reference	
Yes	2.37(1.67-3.27)	<0.01	0.93(0.59-1.41)	0.74
**Chemotherapy**				
No/unknown	Reference		Reference	
Yes	1.35(1.19-1.54)	<0.01	1.50(1.28-1.75)	<0.01
**Sur (pri)**				
Unilateral	Reference		Reference	
Bilateral or more area	2.41(1.97-2.97)	<0.01	1.45(1.18-1.80)	<0.01
No/unknown	10.59(8.67-13.04)	<0.01	3.15(2.50-4.00)	<0.01
**T stage**				
T1	Reference		Reference	
T2	4.11(3.06-5.56)	<0.01	2.11(1.55-2.90)	<0.01
T3	6.71(5.28-8.67)	<0.01	2.49(1.91-3.29)	<0.01
Not stated	17.67(13.73-23.07)	<0.01	4.05(3.04-5.46)	<0.01
**N stage**				
N0	Reference		Reference	
N1	2.49(2.20-2.83)	<0.01	1.90(1.64-2.20)	<0.01
Not stated	3.12(2.72-3.58)	<0.01	1.27(1.08-1.49)	<0.01
**CA-125**				
Negative	Reference		Reference	
Positive	6.20(4.29-9.41)	<0.01	2.22(1.51-3.42)	<0.01
Borderline/unknown	4.77(3.25-7.33)	<0.01	1.87(1.25-2.91)	<0.01
**Bone Mets**				
No/unknown	Reference		Reference	
Yes	10.68(8.18-13.88)	<0.01	3.33(2.44-4.51)	<0.01
**Brain Mets**				
No/unknown	Reference		Reference	
Yes	16.26(10.11-26.08)	<0.01	5.72(3.19-10.28)	<0.01
**Liver Mets**				
No/unknown	Reference		Reference	
Yes	7.62(6.70-8.65)	<0.01	3.64(3.17-4.18)	<0.01

**Abbreviations**: OR=odd ratio; 95%CI=95% confidence intervals; Sur (pri)=surgical treatments on primary site; CA-125=cancer antigen 125; Mets=metastasis.

**Table 4:** The prognostic factors for overall survival in ovarian cancer with lung metastasis by Cox regression analyses.

Subject Characteristics	Univariable		Multivariable	
	HR (95%Cl)	P-value	HR (95%Cl)	P-value
**Age at diagnosis**				
18-49	Reference		Reference	
50-69	1.04(0.85-1.26)	<0.01	1.02(0.83-1.25)	0.85
70-79	1.36(1.10-1.69)	<0.01	1.14(0.91-1.42)	0.25
≥80	2.22(1.77-2.78)	<0.01	1.34(1.05-1.71)	0.02
**Race**				
Black	Reference		Reference	
White	0.83(0.69-0.99)	<0.01	0.96(0.79-1.17)	0.71
Other/not stated	0.68(0.53-0.88)	<0.01	0.79(0.60-1.03)	0.08
**Marital status**				
Married	Reference		Reference	
Unmarried	1.42(1.25-1.60)	<0.01	1.10(0.97-1.26)	0.12
Unknown	1.53(1.12-2.10)	<0.01	0.86(0.62-1.20)	0.37
**Histology**				
Adenocarcinoma	Reference		Reference	
Serous adenocarcinoma	0.60(0.53-0.69)	<0.01	0.84(0.72-0.98)	0.03
Others/not stated	1.23(1.04-1.45)	<0.01	1.05(0.88-1.25)	0.60
**Grade**				
Well differentiated	Reference		Reference	
Moderately differentiated	2.42(0.75-7.83)	<0.01	3.96(1.21-12.96)	0.02
Poorly differentiated	2.34(0.75-7.31)	<0.01	3.54(1.12-11.23)	0.03
Undifferentiated	2.02(0.64-6.34)	<0.01	3.43(1.08-10.93)	0.04
Unknown	3.43(1.10-10.65)	<0.01	2.68(0.85-8.47)	0.09
**Regional nodes examined**				
0	Reference		Reference	
1-10	0.53(0.44-0.64)	<0.01	0.88(0.70-1.10)	0.25
11+	0.48(0.38-0.62)	<0.01	0.87(0.66-1.14)	0.30
Not stated	1.32(1.02-1.72)	<0.01	0.89(0.67-1.19)	0.44
**Radiotherapy**				
No/unknown	Reference		Reference	
Yes	2.02(1.45-2.83)	<0.01	1.09(0.74-1.59)	0.67
**Chemotherapy**				
No/unknown	Reference		Reference	
Yes	0.25(0.22-0.29)	<0.01	0.36(0.31-0.43)	<0.01
**Sur (pri)**				
Unilateral	Reference		Reference	
Bilateral or more area	1.04(0.81-1.34)	<0.01	1.01(0.78-1.31)	0.92
No/unknown	3.12(2.44-3.98)	<0.01	2.20(1.66-2.91)	<0.01
**T stage**				
T1	Reference		Reference	
T2	1.10(0.78-1.55)	<0.01	1.10(0.77-1.56)	0.60
T3	0.82(0.61-1.09)	<0.01	0.99(0.73-1.34)	0.96
Not stated	1.60(1.19-2.16)	<0.01	1.06(0.78-1.44)	0.72
**N stage**				
N0	Reference		Reference	
N1	0.97(0.84-1.11)	<0.01	1.09(0.94-1.27)	0.26
Not stated	1.60(1.38-1.85)	<0.01	1.10(0.93-1.30)	0.25
**CA-125**				
Negative	Reference		Reference	
Positive	0.88(0.56-1.39)	<0.01	1.65(1.03-2.66)	0.04
Borderline/unknown	1.35(0.85-2.16)	<0.01	1.69(1.04-2.74)	0.03
**Bone Mets**				
No/unknown	Reference		Reference	
Yes	1.75(1.40-2.21)	<0.01	1.22(0.95-1.56)	<0.01
**Brain Mets**				
No/unknown	Reference		Reference	
Yes	3.16(2.23-4.49)	<0.01	1.91(1.27-2.88)	0.12
**Liver Mets**				
No/unknown	Reference		Reference	
Yes	1.32(1.18-1.50)	<0.01	1.25(1.09-1.42)	<0.01

Abbreviations: HR=hazard ratio; 95%CI=95% confidence intervals; Sur (pri)=surgical treatments on primary site; CA-125= cancer antigen 125; Mets=metastasis.

## Abbreviations

AUC: area under the curve
CA-125: cancer antigen 125
CICs: clinical impact curves
C-index: concordance index
DCAs: decision curve analyses
LM: lung metastases
OC: ovarian cancer
OS: overall survival
PSM: Propensity score matching
SEER: Surveillance, Epidemiology, and End Results
